# Reliability of self-reported career histories reported by former professional association football players included in the health and ageing data iN the game of football (HEADING) study

**DOI:** 10.1093/annweh/wxag032

**Published:** 2026-05-14

**Authors:** Ioannis Basinas, Helena Copsey, Fiona S Carson, Finlay Brooker, Steven Robertson, Yvonne van Hoecke, Simon Kemp, Giulia Seghezzo, Neil Pearce, Valentina Gallo, Damien M McElvenny, John W Cherrie

**Affiliations:** Centre for Occupational and Environmental Health, Division of Population Health, Health Services Research and Primary Care, School of Health Sciences, Faculty of Biology, Medicine and Health, The University of Manchester, Oxford Road, Manchester M13 9PL, United Kingdom; Science, Insights and Influence, Institute of Occupational Medicine, Pentland Science Park, Bush Loan, Penicuik EH26 0PL, United Kingdom; Science, Insights and Influence, Institute of Occupational Medicine, Pentland Science Park, Bush Loan, Penicuik EH26 0PL, United Kingdom; Science, Insights and Influence, Institute of Occupational Medicine, Pentland Science Park, Bush Loan, Penicuik EH26 0PL, United Kingdom; Science, Insights and Influence, Institute of Occupational Medicine, Pentland Science Park, Bush Loan, Penicuik EH26 0PL, United Kingdom; Department of Medical Statistics, London School of Hygiene & Tropical Medicine, Keppel Street, London WC1E 7HT, United Kingdom; Department of Medical Statistics, London School of Hygiene & Tropical Medicine, Keppel Street, London WC1E 7HT, United Kingdom; Rugby Football Union, Twickenham TW2 7BA, United Kingdom; Department of Non-Communicable Disease Epidemiology, London School of Hygiene & Tropical Medicine, Keppel Street, London WC1E 7HT, United Kingdom; Department of Medical Statistics, London School of Hygiene & Tropical Medicine, Keppel Street, London WC1E 7HT, United Kingdom; Department of Sustainable Health, Campus Fryslan, University of Groningen, Wirdumerdijk 34, 8911 CE Leeuwarden, The Netherlands; Department of Epidemiology, University Medical Centre Groningen, PO Box 30.001, 9700 RB Groningen, The Netherlands; Centre for Occupational and Environmental Health, Division of Population Health, Health Services Research and Primary Care, School of Health Sciences, Faculty of Biology, Medicine and Health, The University of Manchester, Oxford Road, Manchester M13 9PL, United Kingdom; Science, Insights and Influence, Institute of Occupational Medicine, Pentland Science Park, Bush Loan, Penicuik EH26 0PL, United Kingdom; Science, Insights and Influence, Institute of Occupational Medicine, Pentland Science Park, Bush Loan, Penicuik EH26 0PL, United Kingdom; School of Engineering and Physical Sciences, Heriot Watt University, Edinburgh EH14 4AS, United Kingdom

**Keywords:** repetitive sub-concussive head impacts, heading, professional soccer, validation, playing careers

## Abstract

**Aim:**

To assess the ability of former professional association football players in England to recall their playing careers.

**Methods:**

Self-reported data regarding playing careers were available from former professional football players participating in the Health and Ageing Data IN the Game of football (HEADING) study. We compared the self-reported data from 141 participants regarding the teams, positions, periods, and leagues they played for against the data available in publicly available records, compiled with the assistance of the English Professional Footballers’ Association.

**Results:**

Self-reporting of career histories occurred a mean of 28.5 years since the players had their last competitive season. Overall, 20 discrepancies between self-reported and register-based data were observed in the career histories of 18 (12.8%) individual players. This is in line with data from earlier reliability studies on the self-reporting of occupational histories. The most common error observed was incorrect recording of years of play in certain clubs, occurring in 9 instances. Missing teams in self-reports were observed in another 8 cases.

**Conclusion:**

The results suggest that former professional association football players can provide plausible information over long periods regarding the general characteristics of their professional career. This information could form the basis for a reliable exposure assessment within epidemiological analyses. Further work is required to assess recall of the amount of heading and other head impacts during training and play.

What's important about this paper Recall bias is an important potential limitation when assessing historical exposures within epidemiological studies. The present study examined the ability of former professional football players to recall their playing histories against publicly available records between 6 and 56 years after the end of their careers. Overall, the results show that former professional football players can provide plausible information related to the basic characteristics of their professional playing history.

## Introduction

Repetitive sub-concussive head impacts (RSHIs) in professional association football (soccer) are blows to the head from heading footballs, contacts from other players with the head, or contacts between the head and any other objects (eg goal posts) during play (Gysland et al. 2012). There is suspicion that high cumulative exposure to RSHIs, for example, associated with a professional soccer playing career, may damage a player's neurological health, although the available evidence to date is somewhat limited due to poorly defined data on RSHIs in epidemiological studies (Mainwaring et al. 2018). A recent meta-analysis of 4 case–control and 5 cohort studies among former professional footballers (Howarth et al. 2025) showed that participation in professional football was associated with increased odds of neurodegenerative disease, including dementia and motor neuron disease. However, it remains possible that there are alternative explanations for the observed associations, other than the blows to the head, such as the high level of physical exercise required of professional athletes (Julian et al. 2021).

In most neurodegenerative diseases, a long latency period between prolonged and repeated exposures and symptoms onset is observed. Consequently, studying the association between an exposure, eg RSHI, and a neurodegenerative disease (eg dementia) is most frequently based on indirect surrogates of the exposure (eg profession as a player or position played). This has also been the case in recent studies of former professional football players (Chiò et al. 2009; Mackay et al. 2019; Śmigielski et al. 2020; Russell et al. 2021). Accurate and reliable assessment of historical exposures is vital for drawing valid inferences related to disease causality, and for the identification of exposure-response relationships and quantification of risks (Nieuwenhuijsen 2003). It is well-known that epidemiological studies that rely on subjective exposure assessment methods, such as self-reports and expert opinions, have inherent limitations. Such studies have potential for recall bias (especially when exposures are self-reported) and/or bias because of limited specific knowledge when assessments are performed by experts (Teschke 2003).

As part of the Health and Ageing Data IN the Game of football (HEADING) study, we have recently published an exposure model that can be used to estimate the exposure of individual professional players in England to repetitive sub-concussive head impacts (RSHI) (Basinas et al. 2023; Gallo et al. 2024). For this, we used self-reported playing career data, together with retrospective estimates of the amount of heading and other impacts footballers experience during play and training for each period of the player's career, including periods of youth, amateur, and semi-professional play. The final exposure assessment model showed that exposure to RSHI depends on the playing position, league; decade started play; context (ie match or training); and age (Basinas et al. 2023).

Since data on player careers were based on self-reports obtained during in-person or remote interviews, 1 aspect of the external validation of the developed exposure models was the extent to which the involved players were able to recollect their playing careers. In the present study we describe an evaluation of the players’ ability to recall their playing careers against publicly available records. Our hypothesis was that there will be no difference between the playing histories reported by the players themselves and those available in the public records.

## Materials and methods

The developed exposure models, as well as the collection of playing histories within the HEADING study, have been described (Basinas et al. 2023). Briefly, recruitment of former professional players in England aged over 50 took place between 2019 and 2021. The initial approach (to maintain confidentiality) was by the Professional Footballers’ Association via post and email. A total of 212 people agreed to participate, of whom 190 were interviewed (initially in-person, but remotely during the COVID-19 pandemic). Of these, 159 provided complete playing histories that included estimates of impact frequencies from heading and other sources. Playing histories were based on self-reports as part of the interview.

The identities of those interviewed were provided by colleagues from the London School of Hygiene and Tropical Medicine for this specific validation exercise. Self-reported playing careers were compared to those published on the Barry Hugman's Footballers (BHF) online reference resource (https://barryhugmansfootballers.co.uk/) supplemented by records available in Wikipedia (https://en.wikipedia.org). The BHF resource contains records for every footballer from 1946 to 2015. The resource is an extension of a series of books, later compiled in a single publication (Hugman 2015), authored by Barry Hugman and published with support from the English Professional Footballers’ Association.

For the analysis, we first compared the reporting of the professional and semi-professional clubs played for, both in terms of completeness and the order in which the players played. We also looked on the years played per club and overall, and how different these were from the official records. For comparisons between continuous and categorical variables, Student t-tests and chi-square tests were used, respectively. Ethical clearance was obtained via a minor amendment to the ethical clearance for the HEADING study from the London School of Hygiene and Tropical Medicine's Ethics Committee.

## Results

From the available player data, we were able to link and validate the careers of 141 (89%) players. Demographic and playing history characteristics were similar comparing the validation with the original sample (Table 1). Self-reporting of career histories occurred a mean of 28.5 years since players had their last competitive season.

**Table 1:** Descriptive characteristics of the players included in the present analysis and the overall population used in the development of the exposure models of basinas et al. (Basinas et al. 2023).

Characteristic	Players in present analysis	Players in original analysis
Number of players	141	159
Age of players, mean (SD)	63.1 (8.9)	62.8 (8.7)
Career duration as professional in years, mean (SD)	16.4 (5.0)	16.8 (5.9)
Number of periods played^a^, mean (SD)	6.0 (2.9)	6.0 (3.0)
Position played		
Defender	57 (40.4)	66 (41.5)
Forward	33 (23.4)	38 (23.9)
Goalkeeper	5 (3.6)	6 (3.8)
Midfield	42 (29.8)	44 (27.8)
Utility^b^	4 (2.8)	5 (3.1)
Years since last competitive season as player	28.2 (10.2)	27.8 (10.3)

^a^Period of play refer to individual playing contract. During data collection, participants were asked to fill out each contract and team separately.

^b^Utility players are players frequently playing in more than one position.

A total of 18 (12.8%) players reported playing histories that were found to contain discrepancies with the available records. Twenty distinct discrepancies in the questionnaire data of those players were identified (Table 2).

**Table 2:** Results from comparisons of the player self-reported career histories against the records available on the barry hugman's footballers reference (Hugman 2015).

Discrepancy in self-reported data	N (%)
One club missing	4 (2.8)
Two clubs missing	2 (1.4)
Three or more clubs missing	2 (1.4)
One or more club out of order	1 (0.7)
Years of play recorded wrong for one club	3 (2.1)
Years of play recorded wrong for two clubs	2 (1.4)
Years of play recorded wrong for three or more clubs	4 (2.8)
One or more clubs included where player did not appear/play in any games	1 (0.7)
Playing position incorrect	1 (0.7)

The records of 141 players were compared. Overall, 20 discrepancies were observed over the careers of 18 individual players.

The most common discrepancy observed was incorrect recording of years of play in certain clubs, occurring in nine instances. These included years being recorded incorrectly for a single club (n = 3), for 2 clubs (n = 2), and for 3 or more clubs (n = 4). However, deviations in reported calendar years of play were commonly (67%) within a range of 1 to 2 years before or after the actual period as reported in the records. Another prevalent discrepancy was the exclusion of clubs in players’ responses (n = 8), but in 1 instance, this reflected instructions provided to the participants during interviews to ignore playing periods in teams with a duration <3 months.

Other, less common, misreports by the players included an incorrect order of clubs (n = 1), clubs being included in which the player did not participate in any first team games (n = 1), and playing position being incorrect (n = 1).

## Discussion

In the present study, we examined the ability of former professional football players to recall their playing histories between 6 and 56 years after the end of their careers. The study compared self-reported data on the teams, leagues, position, and years they played against publicly available records. Overall, for the vast majority (87.2%) of the interviewed players, we observed no discrepancies in the recollection of their careers and relevant characteristics. For the remaining 12.8% discrepancies, were limited accounting on average for less than 6.5% of the relevant individual responses. This suggests that former professional football players can provide plausible information regarding the basic characteristics of their professional playing history. Given their low prevalence and small deviations in terms of reported calendar years within periods (typically 1-2 years per period), it is unlikely that the presence of these discrepancies would impact the exposure estimates for the HEADING study, which were based on group-based assignment approaches. Use of group-based exposure assignment further implies that any classical measurement error present was transformed to Berkson error (Armstrong 1998). Indeed, when models were repeated with an updated version of the dataset that accounted for the observed disparities, estimated effects remained robust both in effect size and direction (online supplement, Table S1). Consequently, any impacts on the resulting cumulative exposure estimates in the epidemiological analysis would have also been minimal.

Several limitations should be considered when interpreting these findings. The small sample size and number of observed discrepancies (n = 20) may have impacted on our ability to analyze recall with a high precision. In addition, although treated as a gold standard publicly available records, such as the BHF, can themselves contain inaccuracies or omissions, potentially therefore introducing misclassification in our analysis. It is also possible that participants consulted publicly available sources (eg Wikipedia) to assist their recall, and some referred to the presence of those records about themselves during interviews. This may have influenced agreement with the reference data and potentially led to an overestimation of recall accuracy in our analysis. Moreover, the presence of non-differential misclassification and/or of selection bias cannot be totally excluded. Recall accuracy could vary according to individual characteristics (eg, career length or prominence) and participants not included into the analysis due to missing or incomplete records may differ systematically from those included. Finally, some discrepancies may reflect misinterpretation rather than true reporting errors, and unmeasured factors could confound the relationship between RSHI exposure and reporting discrepancies.

Several factors can affect recall ability in self-reported studies, including the level of complexity of the information requested, the formulation of the question used, the duration and importance of the of the events to the participant, and the recall period (Stull et al. 2009). This is even more relevant when research participants are older and may present with varying level of cognitive decline; 23 (16%) of the original sample were assessed to have a mini-mental state examination (MMSE) ≤ 25 (Folstein et al. 1975). Only 4 out of the 18 participants with discrepancies in their playing histories had MMSE scores below that level. Albeit the small numbers, when we compared the prevalence of participants with MMSE ≤25 among those with and without discrepancies in their records, we observed no statistically significant differences (25% vs 12%; chi-square *P*-value: 0.16). When it comes to job histories, previous research has shown that the ability of workers to adequately recall their job history may also be affected by the overall number of jobs held and the duration of employment involved in each job (Teschke et al. 2002; Sonnega et al. 2024). Despite this, the literature suggests that individuals can recall basic attributes of their job history relatively well, including parameters such as their employer, their job title and the start and end dates (ie duration) of their employment. For such parameters, the reported raw agreements in earlier reliability studies comparing self-reports mostly against employer and public records have been reported to be very high, in the range of 70 to 90% (Teschke et al. 2002). Our results of total discrepancies in 12.8% of the evaluated careers are towards the upper end this range.

Concerning association football, only a few studies have previously examined the ability of adult players to recall attributes of the play and these have focused exclusively on the reliability of self-reported estimates of the frequency of head impacts per game (Rutherford and Fernie 2005; Catenaccio et al. 2016; Rodrigues et al. 2019). Such estimates are required for the calculation of the cumulative heading frequency, a suggested appropriate measure for analysis of exposure response relationships in epidemiological studies (Basinas et al. 2022). In general, these studies compared the short-term (ie few weeks/months) recall of the frequency of heading among young active football players against observations made by trained researchers. Comparisons showed strong correlation (typically r > 0.7) between the observed and reported values with either no difference (Rodrigues et al. 2019) or with the presence of some overestimation in the reported values, at least among some participant groups (eg females) (Rutherford and Fernie 2005; Catenaccio et al. 2016). We did not include the frequency of reported headers into our validation because of the lack of a gold standard comparator. The above findings may not necessarily apply to the recall of subjects who played many decades in the past such as the ones included in our study. This is because of differences between these studies and ours in context of parameters assessed, recall intervals and the potential presence of age-related cognitive decline effects in our population (mean age of participants with discrepancies vs those without was 67.2 vs 62.6 years). A direct validation exercise on the accuracy of the reported and modelled head impact number estimates within our study against video recordings of historical games is however within the scope of our future research.

To our knowledge, this is the first study examining the long-term recall of former professional association football players of their playing careers. The results suggest that the players can provide plausible information regarding the teams, leagues, positions, and years they played, which can form a good basis for a reliable exposure assessment to be used in analysis with health data. However, their ability to adequately recall in the long-term important details, such as the average frequency of heading per game, remains unknown.

## Supplementary Material

wxag032_Supplementary_Data

## Data Availability

Data are not publicly available due to the privacy protection of participants. We will consider sharing of pseudonymised data to external researchers, on request for additional analyses meeting the scientific and ethical standards under which the data was collected, and consistent with the overall aims of the project.
