# Supplementary material for: Reliability of self-reported career histories reported by former professional association football players included in the health and ageing data iN the game of football (HEADING) study
Source: Ann Work Expo Health. 2026 May 14;70(4):wxag032. doi: 10.1093/annweh/wxag032 (PMC13174944; doi:10.1093/annweh/wxag032)
Supplement: wxag032_Supplementary_Data [file wxag032_supplementary_data.pdf]

Online Supplement

Reliability of self-reported career histories reported by former professional association football players included in the Health and Ageing Data IN the Game of football (HEADING) study

Ioannis Basinas[a, b], Helena Copsey[b], Fiona S Carson[b], Finlay Brooker[b], Steven Robertson[c], Yvonne van Hoecke[c], Simon Kemp[d], Giulia Seghezzo [e], Neil Pearce[c], Valentina Gallo[f,g], Damien M McElvenny [a,b], John W Cherrie[b,h]

[a]Centre for Occupational and Environmental Health, Division of Population Health, Health Services Research and Primary Care, School of Health Sciences, Faculty of Biology, Medicine and Health, The University of Manchester, Manchester, UK

[b]Science, Insights and Influence, Institute of Occupational Medicine, Edinburgh, UK

[c]Department of Medical Statistics, London School of Hygiene & Tropical Medicine, London, UK

[d]Rugby Football Union, London, UK

[e]Department of Non-Communicable Disease Epidemiology, London School of Hygiene & Tropical Medicine, London, UK

[f]Department of Sustainable Health, Campus Fryslan, University of Groningen, Leeuwarden, The Netherlands

[g] Department of Epidemiology, University Medical Centre Groningen, Groningen, The Netherlands

[h]School of Engineering and Physical Sciences, Heriot Watt University, Edinburgh, UK

Corresponding Author:
Dr Ioannis Basinas: Centre for Occupational and Environmental Health, Division of Population Health, Health Services Research and Primary Care, School of Health Sciences, Faculty of Biology, Medicine and Health, The University of Manchester, Manchester, M13 9PL UK. Ioannnis.basinas@manchester.ac.uk

Table S1. Ssensitivity analysis results of the effects of playing characteristics on the average number of reported headers per training or play event, showing estimates from the original (Basinas et al., 2023) and modified by the publicly available records data.

**Parameter**	***Original data***			***Modified data by publicly available records***		
	β	***e***	***p***	***β***	***e***	***p***
Intercept	2.73	0.12	<.0001	2.73	0.12	<.0001
Position						
Forward/utility	-0.18	0.13	0.15	-0.18	0.13	0.15
Goalkeeper	-3.93	0.38	<.0001	-3.93	0.38	<.0001
Midfield	-0.40	0.12	<0.001	-0.40	0.12	<0.001
Defender	Ref			Ref		
League						
Reserves/Youth	-0.25	0.15	0.10	-0.24	0.15	0.10
Amateur	-0.46	0.14	0.001	-0.46	0.14	0.001
Semi-professional (Leagues < league 2)	-0.22	0.10	0.03	-0.21	0.10	0.03
League one or two	-0.04	0.09	0.66	-0.04	0.09	0.68
Championship	0.05	0.10	0.62	0.05	0.10	0.62
Overseas leagues	-0.28	0.18	0.13	-0.28	0.18	0.13
Premier league	Ref			Ref		
Decade of play (starting)						
≤1950	0.27	0.25	0.29	0.26	0.26	0.23
1960	0.30	0.13	0.02	0.30	0.13	0.02
1970	0.10	0.08	0.20	0.10	0.08	0.20
1990	-0.09	0.07	0.23	-0.09	0.07	0.23
≥2000	-0.29	0.13	0.02	-0.30	0.13	0.02
1980	Ref			Ref		
Context						
Training	-0.41	0.05	<.0001	-0.41	0.05	<.0001
Match	Ref			Ref		
Age (continuous)	-0.04	0.01	<.0001	-0.04	0.01	<.0001

n = number of observations with specific characteristic; β = regression coefficient for log-transformed head impact (header) data; e = standard error; p = p-value.

References

Basinas I, McElvenny DM, Brooker F, Robertson S, van Hoecke Y, Kemp S, Pearce N, Gallo V, Cherrie JW. (2023) Exposure assessment for repeated sub-concussive head impacts in soccer: The HEalth and Ageing Data IN the Game of football (HEADING) study. Int J Hyg Environ Health; 253: 114235.
